# Effectiveness of a multi-level implementation strategy for ASD interventions: study protocol for two linked cluster randomized trials

**DOI:** 10.1186/s13012-018-0757-2

**Published:** 2018-05-09

**Authors:** Lauren Brookman-Frazee, Aubyn C. Stahmer

**Affiliations:** 1 Department of Psychiatry, University of California, San Diego, 9500 Gilman Drive, La Jolla, CA 92093-0812 USA; 2 Child and Adolescent Services Research Center, 3020 Children’s Way MC 5033, San Diego, CA 92123 USA; 3 Department of Psychiatry and Behavioral Sciences, University of California, Davis, UC Davis MIND Institute, 2825 50th Street, Sacramento, CA 95817 USA

**Keywords:** Implementation strategy, Leadership, Provider training, Fidelity, Autism, Mental health services, Education services

## Abstract

**Background:**

The Centers for Disease Control (2018) estimates that 1 in 59 children has autism spectrum disorder, and the annual cost of ASD in the U.S. is estimated to be $236 billion. Evidence-based interventions have been developed and demonstrate effectiveness in improving child outcomes. However, research on generalizable methods to scale up these practices in the multiple service systems caring for these children has been limited and is critical to meet this growing public health need. This project includes two, coordinated studies testing the effectiveness of the ***T****ranslating* ***E****vidence-based Interventions (EBI) for* ***A****SD:* ***M****ulti-Level Implementation* ***S****trategy* (TEAMS) model. TEAMS focuses on improving implementation leadership, organizational climate, and provider attitudes and motivation in order to improve two key implementation outcomes—provider training completion and intervention fidelity and subsequent child outcomes. The TEAMS Leadership Institute applies implementation leadership strategies and TEAMS Individualized Provider Strategies for training applies motivational interviewing strategies to facilitate provider and organizational behavior change.

**Methods:**

A cluster randomized implementation/effectiveness Hybrid, type 3, trial with a dismantling design will be used to understand the effectiveness of TEAMS and the mechanisms of change across settings and participants. Study #1 will test the TEAMS model with AIM HI (*An Individualized Mental Health Intervention for ASD*) in publicly funded mental health services. Study #2 will test TEAMS with CPRT (*Classroom Pivotal Response Teaching*) in education settings. Thirty-seven mental health programs and 37 school districts will be randomized, stratified by county and study, to one of four groups (Standard Provider Training Only, Standard Provider Training + Leader Training, Enhanced Provider Training, Enhanced Provider Training + Leader Training) to test the effectiveness of combining standard, EBI-specific training with the two TEAMS modules individually and together on multiple implementation outcomes. Implementation outcomes including provider training completion, fidelity (coded by observers blind to group assignment) and child behavior change will be examined for 295 mental health providers, 295 teachers, and 590 children.

**Discussion:**

This implementation intervention has the potential to increase quality of care for ASD in publicly funded settings by improving effectiveness of intervention implementation. The process and modules will be generalizable to multiple service systems, providers, and interventions, providing broad impact in community services.

**Trial registration:**

This study is registered with Clinicaltrials.gov (NCT03380078). Registered 20 December 2017, retrospectively registered.

## Background

### Caring for children with ASD is a significant public health concern

**Fig. 1:**
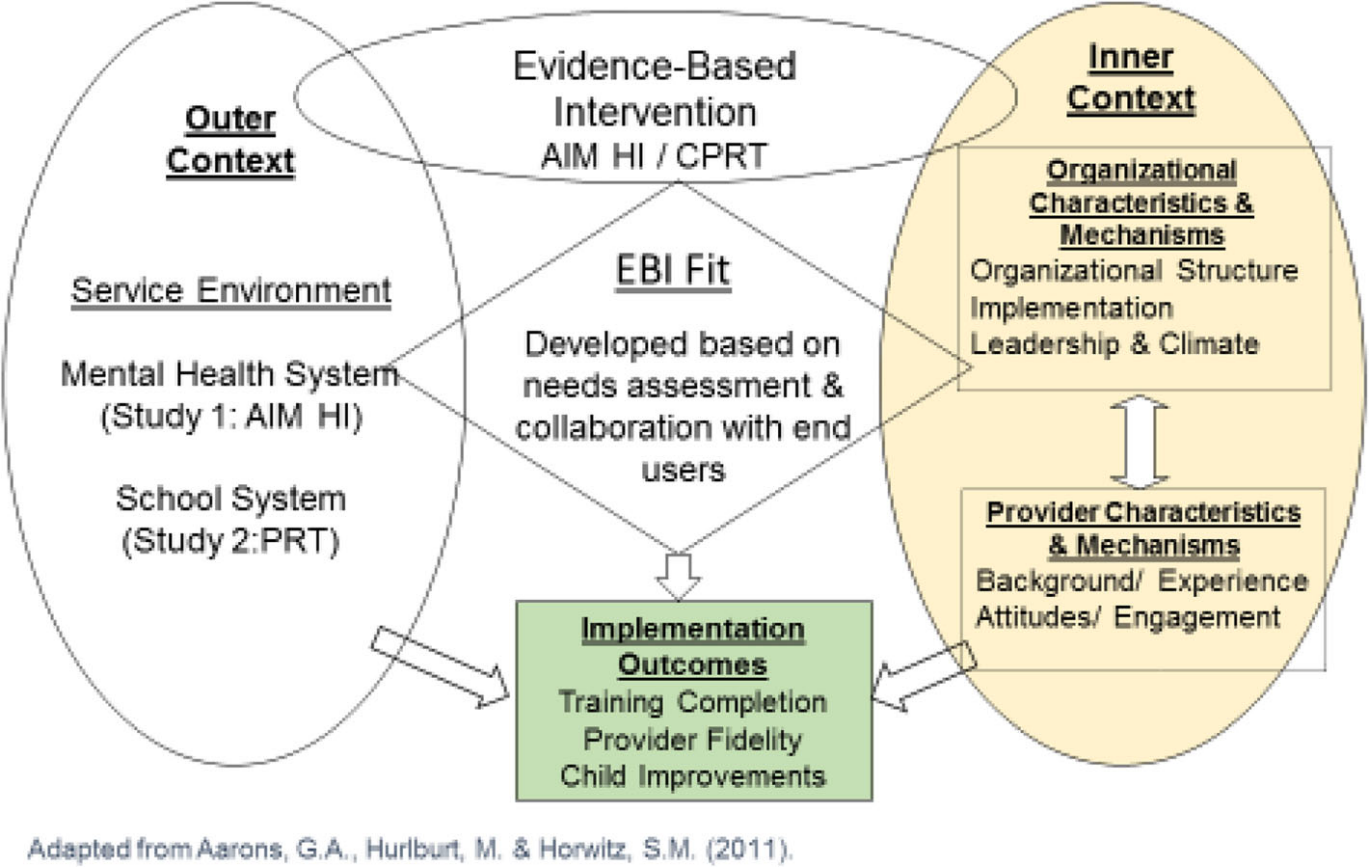
Applying the exploration, preparation, implementation, sustainment (EPIS) conceptual model of implementation to ASD EBI

The Centers for Disease Control (CDC) estimates that 1 in 59 children has ASD [1]. Long-term outcomes for this populations are poor [2–5], and the annual cost in the USA estimated to be $268 billion currently and an estimated $461 billion by 2025 given the high expenditures on care and lost productivity for parents and individuals with ASD [6]. Despite costly investments in the development of efficacious behavioral interventions, these ASD evidence-based interventions (EBI) are not routinely used in community-based care [7–10]. In response to this quality gap, there have been urgent calls for the development and testing of implementation interventions to facilitate successful uptake and sustained delivery of EBI across public service systems [11, 12]. We apply the EPIS Implementation Model [13] (see Fig. 1) to frame our current and previous studies because it was developed for a public child-services context and integrates a multi-level framework. We build on our studies of intervention fit and effectiveness in this project which examines an implementation intervention that acts on interrelated inner context variables of implementation leadership, climate and provider attitudes [14].

### Routine services for school-age children with ASD (outer context)

National data indicate that children with ASD are likely to access a variety of public service systems [15, 16]. The two service systems that accessed most often for school-age children with ASD are education and mental health [17]. The number of children with ASD served by schools has grown fivefold from 93,000 in 2000 to 455,000 in 2011 [18]. The education system (ED) is responsible for targeting a wide range of needs that interfere with a child’s ability to benefit from general ED including improving learning skills such as attention and engagement, and core symptoms of ASD. Over 90% of students with ASD are served in public schools in regular or special ED settings [18]. Mental health (MH) services also play an important role in caring for school-age children with ASD for treatment of behavioral and co-occurring psychiatric problems [16]. Approximately 70% of children with ASD meet criteria for at least one additional psychiatric disorder [15, 19]. Furthermore, challenging behaviors (e.g., aggression, noncompliance, self-injury) are commonly associated with ASD and significantly impact child and family functioning [20].

### Efficacy of behavioral interventions for ASD (intervention characteristics)

Behavioral interventions are considered well established for targeting outcomes in many areas (e.g., academic, communication, social, mental health/behavioral) based on independent reviews by the National Autism Center [21] and National Professional Development Center on ASD [22]. Comprehensive packages of intervention strategies (e.g., pivotal response teaching) have been found to improve social communication, play, and engagement [22–32]. Furthermore, function-based positive behavior support strategies effectively reduce challenging behaviors [20–22, 33]. Despite the growing evidence for behavior interventions for a wide range of treatment targets in children with ASD, these interventions are not consistently or effectively used in routine services [8–10].

### Responding to gaps between research and community practice: development of AIM HI and CPRT interventions (EBI/inner context fit)

Unfortunately, there are concerning gaps between EBI and routine care in ED and MH services. For example, a majority of educators report using an “eclectic” treatment approach [10], which is not supported by empirical data [10, 34]. Studies in MH services indicate that therapists use EBI strategies with low intensity and have very limited training in ASD [8, 9]. Even when teachers and MH providers are aware of EBI for ASD and are attempting to use them, they do not do so with high levels of fidelity [35, 36]. Additionally, parents of children with ASD report poorer satisfaction with community services than parents of children with other special needs, further indicating an ongoing need for improving services [37], particularly for racial/ethnic minority families [38, 39].

In response to these concerning gaps between EBIs and routine care, our research groups have used community-partnered approaches to adapt and implement behavioral EBI for delivery in routine care in multiple service systems [40–43]. AIM HI (“An Individualized Mental Health Intervention for ASD” [44]) refers to a package of well-established, evidence-based behavioral strategies designed to reduce challenging behaviors in children served in MH service settings. CPRT (“Classroom Pivotal Response Teaching” [45]) refers to a naturalistic behavioral intervention adapted from pivotal response training (PRT) for use during classroom activities to target social, communication, behavior, and learning skills. Pilot studies and ongoing large-scale effectiveness trials (NIMH R01MH094317; IES R324A140005; R324B070027) conducted in the targeted community service settings show high levels of feasibility, acceptability, and support the overall effectiveness of the clinical interventions for improving child outcomes when providers complete training and deliver the interventions with fidelity [41, 46]. AIM HI and CPRT research share common methods for developing, adapting, and testing interventions in varied community service settings [43] and report similar outcomes, making them ideal for cross-service setting comparison of innovative implementation interventions.

### Need for multi-level implementation interventions targeting inner context factors to improve implementation outcomes (training completion, fidelity, child outcomes)

Recent data from the AIM HI and CPRT large-scale effectiveness trials indicate that, even with systematically adapted interventions, strong community partnership, and best practices in training and consultation, there is variability in training completion and fidelity (e.g., 17–26% of providers participating in our research-based training do not complete training and 27–30% do not meet fidelity criteria) [36]. Because provider fidelity of EBIs is related to child outcomes [47, 48], testing methods of improving implementation outcomes is critical to ensuring positive child-level outcomes when EBIs are implemented in routine care [49, 50].

### Leadership- and provider-level targets of implementation interventions (see Fig. 2)

Glisson and Williams [51] call for carefully designed, multi-level studies testing specific change mechanisms as they affect both leader- and provider-level factors. However, there is limited research examining cross-level mechanisms linking specific implementation interventions to targeted changes in provider behaviors [52]. Preliminary data from AIM HI and CPRT effectiveness studies align with other literature indicating two inner context variables, provider attitudes and engagement in training [11, 53] and implementation leadership/climate and support strategies [54] are associated with successful implementation.

**Fig. 2:**
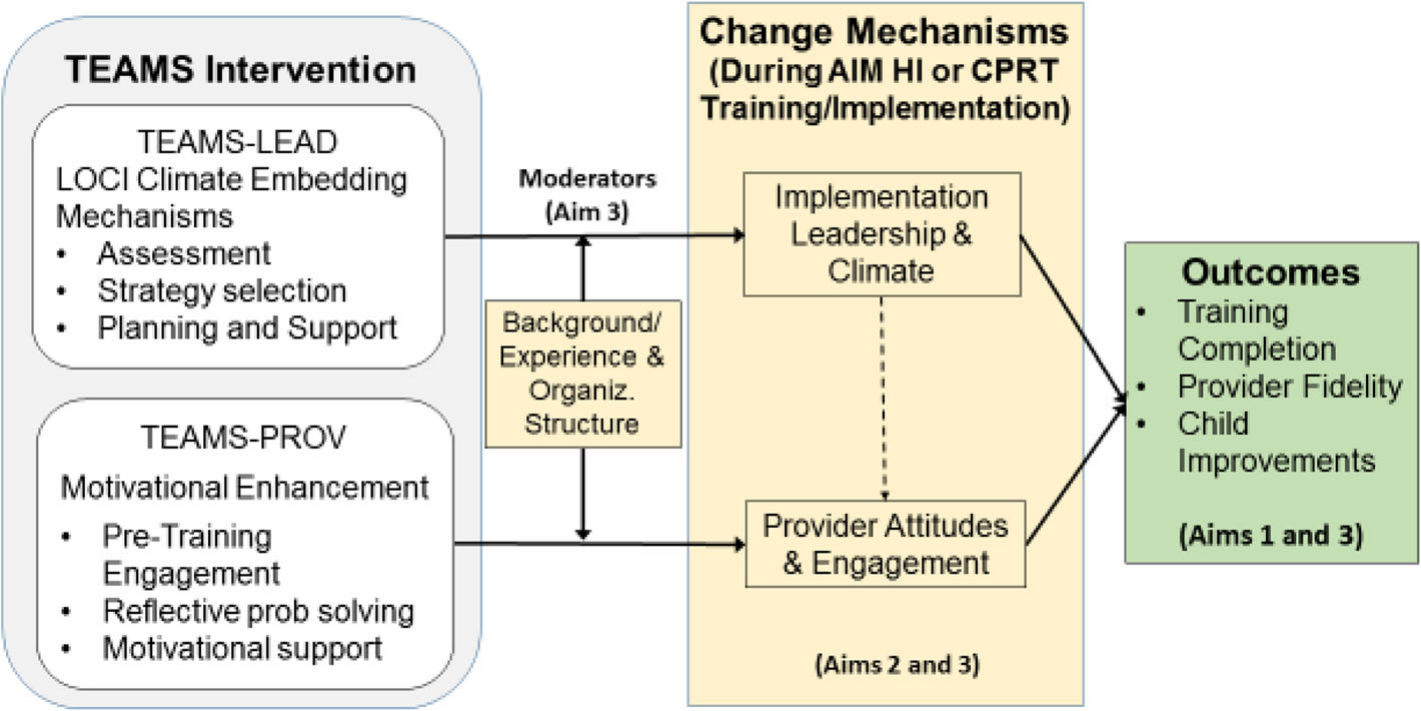
TEAMS intervention, mechanisms, and outcomes

#### Implementation leadership

Recent research highlights the importance of leadership in successful implementation of innovative practices [55–58]. Implementation leadership focuses on specific behaviors and actions that demonstrate the leader’s commitment to, knowledge of, support for, and perseverance during EBI implementation. Implementation leadership can improve the climate for use of EBI [14]. Positive implementation climate and use of support strategies such as training availability and ongoing monitoring of performance has been linked to better sustainment of innovation, improved child outcomes, and decreased staff burnout and turn over [59]. When leaders provide clear guidance during implementation, facilitate support among co-workers and from administration for effective implementation, trainees report an increased sense of competence and satisfaction [60]. Additionally, a clear relationship between organizational culture and climate and child-level outcomes has been identified in MH and ED settings for behavior disorders and ASD [12, 61, 62].

Therefore, the TEAMS Leadership Institute (TLI) module is directed toward a low-cost method of improving implementation climate through specific implementation leadership training [63]. We are focusing specifically on implementation leadership as it is highly targeted (as opposed to more general leadership training) and provides specific EBI strategies that can be used in public service settings with limited resources. TLI draws from the Leadership and Organizational Change for Implementation (LOCI) [54] model which targets development of first-level leaders (e.g., immediate supervisors) with the goal of optimizing EBI implementation [64]. We expect TLI to specifically affect implementation climate, which is related to providers’ perceptions of leadership support of the EBI implementation [65].

#### Provider attitudes

Provider attitudes toward EBI have been linked to practice behavior [66, 67] and have been shown to predict use of EBI [13, 68, 69]. Multiple studies have found that provider attitudes before EBI training, especially openness to the use of EBI and perceptions of EBI appeal, are linked to fidelity after training [13, 70–72]. Furthermore, attitudes toward a specific practice are linked to reported use of that practice [69]. Negative beliefs about a practice may be a barrier to adoption [73, 74]. Data indicate that this may be especially true of behavioral interventions for ASD; however, poor attitudes can be improved with education [75, 76]. These studies, combined with preliminary data from the AIM HI and CPRT effectiveness trials linking attitudes toward ASD EBI and fidelity, indicate that provider attitudes are a promising and important target of implementation interventions aimed to increase uptake.

Therefore, the TEAMS Individual Provider Strategy (TIPS) for training applies well-established motivational enhancement strategies to motivate and engage providers in EBI training. Motivational interviewing (MI) [77–85] is a provider interaction style developed to address ambivalence about behavior change. MI is effective in facilitating behavior change for a wide-range of patient populations, improving treatment participation, attendance, adherence, and patient outcomes in a variety of treatments [86], including behavioral interventions [87]. Although MI is well-established for improving adult and adolescent *client* attitudes and engagement, there is limited application to enhance *provider* EBI training [73]. TIPS uses Miller’s conceptual model and clinical guidelines [88] for MI which emphasize AIM HI and CPRT trainers’ using an empathic learner-centered training style while evoking and strengthening the providers’ motivations for overcoming ambivalence about change (i.e., using AIM HI or CPRT). We will use the well-studied processes of MI in the TIPS module to improve provider attitudes and increase provider engagement in EBI training with the expectation that this will lead to improved EBI training completion, EBI fidelity, and child-level outcomes.

#### Current project

**Fig. 3:**
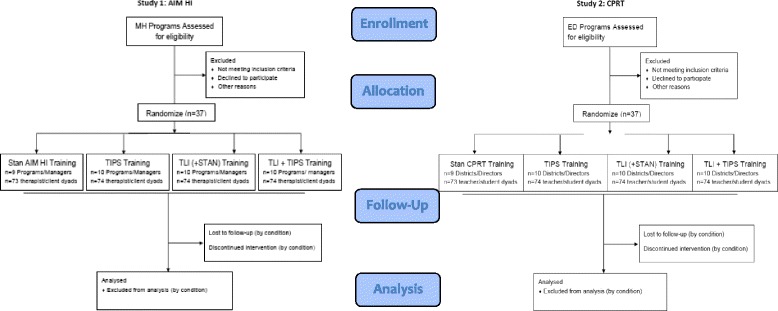
TEAMS consort diagram

This project includes two coordinated studies testing the impact of the “Translating Evidence-based Interventions for ASD: A Multi-Level Implementation Strategy” (TEAMS). TEAMS focuses on improving implementation leadership, organizational climate (TLI), and provider attitudes and engagement (TIPS) in order to improve two key implementation outcomes—training completion and ASD EBI fidelity and subsequent child outcomes. These studies will use a randomized Hybrid implementation/effectiveness, type 3, trial. Study #1 will test the TEAMS model with *An Individualized Mental Health Intervention for ASD* (AIM HI) [44] in publicly-funded mental health services. Study #2 will test TEAMS with *Classroom Pivotal Response Teaching* (CPRT) [41] in school settings (Fig. 3).

A dismantling design will be used to understand the effectiveness of TEAMS and the mechanisms of change across settings and participants [89]. The specific aims and hypotheses are to:

1. Test the effectiveness of the TEAMS modules individually and in combination on implementation outcomes when paired with two ASD EBI. (a) Compared to standard ASD EBI training (control) and individual TEAMS modules (TLI or TIPS), the full TEAMS model (TLI + TIPS) will lead to more positive implementation outcomes for providers (training completion, fidelity) and children (improvements in targeted symptoms).
2. Test the impact of TEAMS modules on organization- and provider-level mechanisms of change. (a) TLI will increase use of implementation leadership strategies, and TIPS will lead to greater changes in provider attitudes and engagement in EBI training.
3. Identify moderators and mediators of implementation outcomes. (a) Identify provider and organization characteristics that moderate implementation outcomes and (b) identify provider- and leader-level mechanisms of change that mediate implementation outcomes.

## Method

This Hybrid, type 3 implementation/effectiveness trial will use a cluster randomized factorial dismantling design to examine the independent and combined effectiveness of TLI and TIPS. MH programs (AIM HI study) and School districts (CPRT study) from San Diego, Sacramento, and LA Counties will be randomized to one of four conditions (STANDARD EBI-specific training only (control condition); TIPS (Motivational Module); TLI (LOCI Implementation Leadership Module) or TIPS+TLI (Combined Modules). Data collection will be coordinated between the two studies and pooled for analyses.

### Sample

The combined multi-level sample for both studies will include 74 programs/districts, 74 agency/district leaders (1 per agency/district), 590 providers (average of 8 per program/district), and 590 children (1 per provider). This sample size was determined so as to be sufficiently powered for aim 3 mediation analyses. (Refer to Table 1 for the breakdown of participants by study and type).

**Table 1:** Participants by type and study

AIM HI study (37 MH programs)	CPRT study (37 school districts)	Combined (74 agencies)
37 Program managers	37 Program specialists	74 Leaders
295 Therapists	295 Teachers	590 Providers
295 Clients	295 Students	590 Children
*627 Total AIM HI*	*627 Total CPRT*	*1254 Total Combined*

### AIM HI study eligibility

Programs are eligible for enrollment if they provide publicly funded outpatient or school-based psychotherapy services to children in the San Diego, Sacramento, or Los Angeles areas. Leaders are eligible if they provide administrative oversight to an enrolled program site. Therapist eligibility includes the following: employed as staff or a trainee in an enrolled program; anticipated to provide services for at least the next 7 months (i.e. practicum or internship not ending in next 7 months); has an eligible child on current caseload; and did not participate in the prior AIM HI effectiveness trial. Families are enrolled as a dyad with a participating therapist. Eligible families have a child aged 5 to 14 years who has a current ASD diagnosis on record.

### CPRT study eligibility

School districts are eligible for enrollment if they provide publicly funded educational services to students in preschool through fifth grade in the San Diego, Sacramento, or Los Angeles areas. Leaders are eligible if they provide administrative oversight at an enrolled district. Teacher eligibility includes the following: employed in an enrolled district; anticipated employment for at least the full training academic year; has an eligible child in his or her classroom; and did not participate in the prior CPRT effectiveness trial. Families are enrolled as a dyad with a participating teacher. Eligible families have a child aged 2 to 10 who has a primary educational classification of ASD.

### Procedures

#### Enrollment procedures

Programs and districts will be recruited in three waves, over 3 years. Once programs are enrolled, leaders from within the enrolled programs/districts will be recruited. Potentially eligible providers will then be recruited through presentations at standing staff meetings or team meetings. Providers will then identity potentially eligible family participants from their caseload or classrooms and will obtain permission for the research team to contact the caregiver to recruit for participation. All participants will be provided information about the study from research staff and provide consent obtained prior to data collection.

## ASD EBI (AIM HI/CPRT) intervention and training procedures

AIM HI and CPRT are manualized programs with user-friendly materials for training, intervention planning, and fidelity monitoring provided in printed and web-based formats. AIM HI is a package of evidence-based parent-mediated and child-focused strategies, designed to reduce behavior problems in children with ASD ages 5 to 13 served in MH programs. AIM HI involves a series of 10 steps (8 required, 2 as indicated) and within-session elements aimed to teach parents of children with ASD to manage challenging behaviors and teach children positive alternative skills (e.g., self-regulation, social skills) using applied behavioral analytic EBI strategies. There are no set number of sessions as the steps can be repeated as needed; however, a minimum of 13 sessions are required. It takes approximately 6 months to accommodate inevitable missed sessions and the variability in the length of time it takes each family/therapist to complete the steps. CPRT is a naturalistic behavioral intervention designed to increase the “pivotal” child responses of *motivation, initiation* and *breadth of attention*. Specific components of CPRT include providing clear and appropriate instructions, sharing control with the child by providing choices and taking turns, interspersing easy and difficult tasks, requiring responding to multiple materials, providing contingent consequences, reinforcing attempts, and providing reinforcement that is directly related to behavior. CPRT has procedures for using components across groups of students and includes specific resources for teachers such as methods for targeting educational goals and curriculum areas using CPRT, data collection materials etc.

### Provider training procedures

The training curricula follow the manual in both cases. Consistent with our previous studies, training and coaching will be conducted by MA or postdoctoral level researchers with extensive training and experience with AIM HI or CPRT. (Refer to Table 2 for a summary of the training components and process.) AIM HI and CPRT have established training plans developed based on the current knowledge of adult learning theory, health care provider behavior change [90, 91], and data from our pilot and effectiveness studies. Although there is variability in scheduling and details based on the needs of the specific provider group (i.e., therapists or teachers), training in both interventions includes active, systematic, direct, and explicit instructional methods (e.g., step-wise training of general principles then specific strategies; interactive, structured performance-based feedback on skill-building; practice with feedback; sustained training efforts) that have been associated with improved provider competence and patient outcomes [90–95]. The inclusion of ongoing coaching/consultation in both models is especially important for improving and maintaining treatment integrity and provider confidence in their ability to use the intervention [96, 97].

**Table 2:** ASD EBI provider training components

	AIM HI	CPRT
Initial didactic training—lecture material, video examples, case illustrations, and hands-on practice with coaching and discussion	12 h (over 2 days)	8 h
Ongoing consultation/coaching—occurs while provider delivers intervention to target child and includes video-based feedback^1^	11 1-h sessions over 6 months (9 group, 2 indiv.)	4 1-h group sessions and 6 individual coaching sessions over 5 months
Total length of training	22 h/6 months	18 h/6 months

^1^Trainers will review videos submitted by providers at least monthly to provide performance feedback during consultation/coaching sessions

### Trainer training procedures

Both ASD EBI have established trainer training processes, which include ongoing fidelity monitoring for trainers by the PIs/intervention developers.

### TEAMS implementation strategy procedures

#### TEAMS Leadership Institute (TLI) module

This module is based on the Strategic Implementation Leadership (which includes implementation support strategies) module of the Aarons’ & Ehrhart LOCI intervention [54]. TLI provides leaders a menu of implementation leadership and climate building strategies and a method for choosing appropriate strategies based on a pre-training assessment. All TLI training and coaching will be audio recorded and a subset of sessions will be coded quarterly to ensure ongoing fidelity. TLI fidelity will be examined across sites during ongoing supervision, and concerns will be readily addressed.

TLI includes 5 key components of LOCI: 1. *Assessment*: The Implementation Leadership Scale (ILS) [98] and Implementation Climate Scale (ICS) [99] will be completed by first-level leaders participating in the intervention, his/her subordinates (i.e., providers), and executive leaders. Data are synthesized into a detailed feedback report and used to develop an implementation leadership plan specific to the leader that will build a positive implementation climate. 2. *Initial training*: TLI begins with a 3-h implementation leadership didactic and interactive session that includes training in implementation leadership and identifying leader behaviors and strategies that can be used to build a climate for EBI implementation. Facilitators and leaders will work together to review the assessment data and identify strengths and areas for development. Leaders will develop a plan for using specific implementation support strategies. 3. *Coaching*: Weekly, brief (15–30 min) coaching calls keep leaders on track with goals and plans. Coaching includes review of progress toward goals, updating plan based on emergent issues, and problem solving. 4. *Follow-up session*: At month 4, leaders attend a 2-h booster session to assess progress and guide modification of the implementation leadership plans. 5. *Graduation*: Graduation is deliberately included in TLI to mark completion of the program. TLI programs will have a group-based graduation for the leaders and provider trainees at EBI training completion to celebrate accomplishments, processes’ changes, and plan for the future.

#### TIPS for training module

The TIPS module applies MI principles and strategies to address attitudinal barriers through elicitation of change talk and improve engagement in training [100]. AIM HI and CPRT trainers (in the TIPS conditions only) will incorporate MI during training and ongoing consultation/coaching with providers using established MI approaches to increase provider engagement and problem solving throughout the training process [101]: 1. *Pre-training call*: Prior to AIM HI or CPRT initial didactic workshops, providers will receive a pre-training call designed to provide information about training and the intervention, and promote change talk around training (What do you anticipate to go well during this training? Why is this training important to you?). 2. *Proactive planning*: During the initial workshop, trainers will elicit change talk about participating in the EBI training. (e.g., “What would you like to accomplish during this training?” “What do you see as the benefits of getting started in this intervention?”). Trainers will have discussions that elicit self-motivational statements about provider plans for using the intervention strategies, attending training, and adhering to the training plan (e.g., “What steps can you to take to include strategy X in this week’s session?”). Trainees will emphasize autonomy and build confidence in the use strategies and problem solving any challenges. Planning worksheets will include affirmations of strengths and change efforts during each session. 3. *On-going reflection*: During each coaching/consultation session, providers will reflect on the importance of the training and their confidence in their use of the strategies at the beginning and end of each session. Trainer feedback will be tailored to link provider behavior to changes in child or parent behavior. Trainers will use reflective methods to facilitate provider independence with the strategies, relate training to broader professional development, and develop possible solutions and next steps for any challenges—sessions will end with collaborative development of next steps. 4. *Motivational notes*: Providers will receive a weekly motivational text or email (depending on preference) to encourage on-going participation. Messages will be based on identified attitudinal barriers for each participant. They might include motivational texts and/or reminders about attendance and practice and therapy outcomes.

TIPS fidelity will be assessed using the 12-item MI Coach Rating Scale (MI-CRS) [102] developed by Dr. Naar-King, the MI expert on the study. Trainers will provide audio recordings of their interactions with providers three times monthly following training and then quarterly thereafter. A similar sample of training sessions for trainers using only standard training (not trained in MI) will be coded to ensure they are not using MI strategies. TIPS fidelity will be examined across sites during ongoing supervision, and concerns will be readily addressed.

### Procedures for randomization and data collection

#### Randomization

Programs and districts will be randomized by an independent researcher and stratified by county and study via computer-generated randomization to one of four conditions per study following official study enrollment

#### TLI

For the 2 groups randomized to TLI or TLI + PROV, the initial TLI workshop will be completed before provider enrollment. This will be done each spring/summer. Each fall, programs, and districts in all conditions will begin provider/child dyad enrollment, complete pre-assessments, and training.

#### Outcome measures

Because we are testing an implementation intervention and our ASD EBI have been shown to be effective in our large-scale effectiveness trials, our primary measures are related to provider implementation (training completion/fidelity). However, consistent with Hybrid, type 3 designs, we will collect child outcome data as secondary outcome measures (within session behavior; standardized measures). Outcomes will be collected for leaders and providers using the following timeframes: Baseline (BL—initial program/district enrollment), Pre (PRE—prior to EBI Training), Post training (POST—6 months after initiation EBI training), and Follow-Up (12 months post PRE). Parents will complete child measures at PRE and POST as children may not remain with the same provider or be receiving services at follow-up. Data will be collected by administering a brief battery of measures (including demographic measures for all participants) via on-line survey or phone interview. The outcome constructs include implementation outcomes (provider and child) as well as measurement of the specific mechanisms of change expected from the implementation intervention. Refer to Table 3 for specific outcome measures and timing.

**Table 3:** Study measures

Purpose (aim)	Construct	Measure/indicators	Timeframe			
			BL	Pre	Post	FU
Outcomes (aim 1)	Training completion	Provider training/consultation completion (based on trainer-rated attendance tracking)			X	
	Provider fidelity	Fidelity of ASD EBI (*also collected mid-training for total of 3 obs.*)		X	X	.
	Child improvements	Child session behaviors (disruptive, engagement)		X	X	
		Child symptom improvement (ECBI [AIM HI]/PDDBI [CPRT])		X	X	
Mechanisms of change (aims 2, 3)	Provider engagement	Training/consultation attendance (trainer rated)			X	
		Adherence to training requirements (video submissions, forms)			X	
	Provider attitudes	Evidence-Based Practice Attitudes Scale (EBPAS)		X	X	X
		Adapted motivation inventory [116]		X	X	X
	Impl. Leadership & Climate	Implementation Leadership Scale (leaders and providers) [98]	X	X	X	X
		Implementation Climate Scale [117] (leaders and providers)	X	X	X	X
	Implementation support use	Assessment of implementation supports strategies	X	X	X	X
Moderators (aim 3)	Background/experience	AIM HI & CPRT Background Questionnaire (leaders & providers)	X (L)	X (P)		
	Org. structure	Program/District Structure Questionnaire	X			

#### Provider fidelity measures

Provider ASD EBI (AIM HI/CPRT) fidelity is the primary study outcome. Provider fidelity will be based on ratings by trained observers blind to study condition and time frame. Fidelity ratings are designed to capture provider use of the core EBI strategies included in the AIM HI and CPRT protocols. Fidelity will be rated with the target child at PRE, mid training, and POST. Fidelity will be coded based on video recordings of sessions/activities using our established procedures for video data collection. AIM HI Fidelity includes 16 Adherence items reflecting expected within-session therapist behaviors in three categories (Structuring Sessions for Skill-Building, Motivating Children, Active Teaching). These are rated separately for parents and children. There are also 4 Effectiveness Items (e.g., session is organized and structured). The inter-rater reliability of these items are high (*M* = .77, range = 0.65 to 0.90 based on 299 double-coded sessions). Furthermore, all individual and composite scores for therapists trained in AIM HI are significantly higher than therapists delivering usual care (based on over 600 coded sessions), providing support for the discriminant validity of the measure. Sessions recordings will be submitted online prior to training and monthly during the 6-month training/consultation period*.* CPRT fidelity includes 15 adherence items reflecting expected within-session teacher behaviors in three categories (maximizing student motivation, facilitating student responding, providing appropriate consequences). There are also 4 effectiveness items (e.g., manages distractions in the teaching environment). All items are rated on a 1–5 Likert scale after observing the complete activity with 1 being teacher does not implement or implements correctly less than 30% of the time to 5, teacher implements correctly throughout the session (100%). The inter-rater reliability of these items are high (*M* = .73, range = 0.50–0.90 based on 240+ double-coded sessions).

#### Coder training

Coders will be trained to code fidelity for one of the EBI (AIM HI or CPRT), and child session behavior. All coders will be blind to treatment groups (STAN, TLI; TIPS; TLI + TIPS) and time frame. They will be trained to 80% reliability across 6 videos before coding for the study. If there is less than 80% agreement between the investigator and the coder, additional training will be provided until agreement is achieved. Twenty-five percent of all video samples will be scored by a second person for the purpose of monitoring reliability of coding.

## Data analysis

### General data analytic plan for aims 1–3

All data analytic strategies will follow the recommendations of Brown et al. and the Prevention Science and Methodology Group for randomized field trials [103]. Preliminary data screening and cleaning will require examination of the data distributions for normality and missing data patterns at both the univariate and multivariate level. Preliminary data analysis will also be conducted to determine if there are significant differences between the two studies (CPRT and AIM HI) on target variables. If no significant differences are found, data from the two studies will be integrated to maximize the available data for specific analyses. In the case that outcome variables differ by study, variables representing study will be included as a covariate in all models. Some of the target outcome variables in the two studies were measured using different scales (e.g., AIM HI and CPRT Fidelity). To ensure comparability on these measures, variables will be converted to *z*-scores before statistical comparisons are made. Hierarchical linear modeling (HLM) will be used as the primary, but not only, statistical model due to the nested structure of the data and when the outcome measure is at the provider/outcome level: repeated measures [level-1] nested within providers/children [level-2] nested within program/district [level-3]). Possible clustering effects will initially be estimated by examining intraclass correlation coefficients to determine if accounting for clustering is necessary. All analyses will use an intent-to-treat approach for the TEAMS groups. Because the TEAMS condition variable will contain four groups, three dummy-coded variables will be created and used as predictor variables in target statistical models. To account for the different type of leader at the program/district level, a level-3 binary variable representing whether or not the agency/district leader is a director or a first-level leader will be used in all analyses. Covariates (e.g., baseline scores) may be included in the models to adjust for nonequivalence at baseline and to minimize bias. All analyses will use AR(1) to model a first-order autoregressive correlation structure for the repeated-measures data. Missing data common to longitudinal designs is readily incorporated in HLM if the data can be assumed to be missing at random. However, because there is no statistical test of data that is missing at random [104], all data analysis will also employ selection models [105] to model relationships of interest for the data missing not at random mechanism, as recommended by Little and Rubin [106]. Of interest in some of proffered hypotheses are the differences in target outcomes between the TEAMS groups over time. The TEAMS groups will be statistically compared when a significant cross-level (Time X TEAMS Group) interaction is evident using the statistical methods of Preacher, Curran, and Bauer [107].

#### Specific analyses for aim 1: test the effectiveness of the TEAMS modules individually and in combination on implementation outcomes when paired with two ASD EBI

To determine if there are significant main effects and interactions between the TEAMS groups on observed therapist fidelity (based on video recorded sessions coded at 3 time points over 6 months), statistical analyses will proceed using the 3-level nested data structure outlined in the General Analytic Plan section. To determine if there are significant differences between the TEAMS groups on observed child behaviors during sessions, statistical analyses will proceed using the 3-level nested data structure outlined in the General Analytic Plan section. To determine if there are differences between the TEAMS groups on child symptoms (EBCI or PDDBI) at 6 months, the 3-level nested data structured will be used.

#### Specific analyses for aim 2: test the impact of TEAMS modules on leader- and provider-level mechanisms of change

Measurement of leader-level outcomes is of primary interest. Implementation Leadership and Climate scores will be aggregated to the leader level. For this reason, HLM models (or single-level regression models if ICC values suggest clustering is negligible) will be used to test for TEAMS group differences on measures of implementation leadership and climate scores at 6 and 12 months. These analyses will control for baseline scores on these measures. To determine if there are significant differences between the TEAMS groups on therapist attitudes (EBPAS), analyses will proceed using the 3-level nested data structure outlined in the General Analytic Plan. These analyses will control for baseline EBPAS scores.

#### Specific analyses for aim 3: identify moderators and mediators of implementation outcomes

Variables representing participant characteristics (e.g., job tenure, gender, race/ethnicity) and organizational characteristics (e.g., size) will be evaluated as moderators of the relationship between the dummy-coded TEAMS group variables and outcome variables specified in aim 1. All analyses will proceed as specified in aim 1 above. Relevant interaction terms between the TEAMS group variables and a given moderator will be created and entered as predictor variables. Two sets of models will be used: one set for provider-level outcomes and a second for child-level outcomes. To examine the hypothesized mediated effects of TEAMS group status on fidelity and child outcomes, multilevel path analysis will be conducted. Measures of provider attitudes, implementation leadership and implementation climate will be tested as mediators in respective models. The specific mediated effects within these models will be tested using the bias-corrected bootstrap approach [108].

### Power analyses

To estimate the sample size needed to detect statistically significant differences between TEAMS groups in the context of the 3-level nested data structure (e.g., aim 1, hypothesis 1), the power program RMASS2 [109] was used. The RMASS2 program is specifically designed to calculate the projected sample size needed for longitudinal data with attrition when a comparison is made between groups. To determine the minimum sample size necessary, several assumptions were made: (1) An alpha level of .05 and a power level of 0.80, (2) an effect size of *d* = .39 (based on the CPRT effectiveness trial ES for impact of training on teacher fidelity), (3) 30% overall attrition rate was specified, and (4) a stationary autoregressive structure (lag 1) was specified for the variance-covariance matrix of the repeated measures, using an autocorrelation value of 0.60. In addition, clustering at the programs/districts level was accounted for via the variance inflation factor (VIF = 1.28): 1 + (*c* − 1) × ICC, where *c* is the average number of providers/children per programs/districts (*n* = 8) and the ICC at the programs/districts level (ICC = 0.04, based on AIM HI therapist fidelity outcome). Given these assumptions, 502 providers/children nested within 63 programs/districts are needed to detect statically significant differences between at least two of the TEAMS intervention groups.

Additional power analyses were conducted to determine the sample size needed to detect a hypothesized mediated effect or moderation of the antecedent to mediator relationship (see Aim 3), which is likely to be the most underpowered effect proposed. This set of power analyses was conducted to determine the minimum necessary sample size to find a statically significant mediated effect from variables presenting the TEAMS groups to a target mediator (e.g., provider attitudes) to a target outcome (e.g., provider fidelity outcome). Using the simulation approach for meditational models of Thoemmes, MacKinnon, and Reiser [110], small effects (*R*^2^ values of 2%) were specified for both the mediator and outcome variables. Accounting for the VIF defined above, 591 providers/children nested within 74 programs/districts are needed to detect statistically significant mediated effect.

## Discussion

AIM HI and CPRT data indicate that (1) implementation leadership/climate and (2) provider attitudes toward EBI are promising targets of implementation interventions. The roles of both factors have been indicated for broader patient populations [54] and also in our current projects. As such, we will apply two, established interventions (LOCI, MI) in the TEAMS model to target these specific mechanisms of change. This study will test the impact of combining standard, EBI-specific training with the two TEAMS modules individually and together on multiple implementation outcomes.

This study builds on a novel, bi-directional, approach to translational research. AIM HI and CPRT were developed in response to a recognized quality gap and stakeholder-identified needs. They were designed specifically for, and in collaboration with, the “end users” of the intervention with the goal of maximizing the “fit” between EBPs and the care settings in which they are delivered [111, 112]. This implementation-effectiveness Hybrid type 3 trial [113] builds on our previous Hybrid Type 1 trials where the primary focus was testing the clinical effectiveness of the ASD EBI.

Testing TEAMS paired with two ASD EBIs, developed for two different service settings offers substantially greater efficiency, generalizability, and reproducibility over testing TEAMS with either EBI alone.

Few studies have systematically examined the mechanisms of action of leader-level interventions and none in ASD [98, 114, 115]. Consistent with RDoC, TEAMS targets potential mechanisms of behavior change at leader and provider levels. The dismantling design will allow us to examine the independent and combined effectiveness of both TEAMS modules and the potential to impact scale-up of EBI across systems. This design is a highly innovative feature that will help us to learn more about common and specific elements of implementation strategies across settings. This is an important innovation as it is essential to understand the potential differential contributions of provider versus leader-level interventions (e.g., At what level and under what conditions are the two targets most impactful in moving implementation outcomes? Is it possible that a provider-level engagement intervention can impact outcomes even in the context of an organization with low implementation leadership or implementation supports?). In addition, examining the moderators will allow for individualization of the TEAMS intervention based on the specific needs of the program, district or provider, increasing efficiency of the intervention.

TEAMS is the first implementation intervention to apply leadership level implementation support and motivational interviewing techniques together to improve EBI training completion, fidelity, and child outcomes. This approach has the potential to improve provider fidelity to the EBI, thereby improving child outcomes.

## Abbreviations

AIM HI: An Individualized Mental Health Intervention for ASD
CPRT: Classroom Pivotal Response Teaching
EBI: Evidence-based intervention
LOCI: Leadership and Organizational Change for Implementation
MH: Mental health
MI: Motivational interviewing
TEAMS: Translating Evidence-based Interventions (EBI) for ASD: Multi-Level Implementation Strategy
TIPS: TEAMS Individual Provider Strategy
TLI: TEAMS Leadership Institute

## References

[1] Baio J, Wiggins L, Christensen DL, et al. Prevalence of Autism Spectrum Disorder Among Children Aged 8 Years — Autism and Developmental Disabilities Monitoring Network, 11 Sites, United States, 2014. MMWR Surveill Summ 2018;67(No. SS-6):1–23. 10.15585/mmwr.ss6706a1. 10.15585/mmwr.ss6706a1 PMC5919599 29701730

[2] Eaves LC, Ho HH (2008). Young adult outcome of autism spectrum disorders. J Autism Dev Disord.

[3] Roux AM, Shattuck PT, Cooper BP, Anderson KA, Wagner M, Narendorf SC (2013). Postsecondary employment experiences among young adults with an autism spectrum disorder. J Am Acad Child Adolesc Psychiatry.

[4] Orsmond GI, Shattuck PT, Cooper BP, Sterzing PR, Anderson KA (2013). Social participation among young adults with an autism spectrum disorder. J Autism Dev Disord.

[5] Shattuck PT, Narendorf SC, Cooper B, Sterzing PR, Wagner M, Taylor JL (2012). Postsecondary education and employment among youth with an autism spectrum disorder. Pediatrics.

[6] Leigh JP, Du J (2015). Brief report: forecasting the economic burden of autism in 2015 and 2025 in the United States. J Autism Dev Disord.

[7] Brookman-Frazee L, Baker-Ericzén M, Stadnick N, Taylor R (2012). Parent perspectives on community mental health services for children with autism spectrum disorders. J Child Fam Stud.

[8] Brookman-Frazee L, Drahota A, Stadnick N, Palinkas LA (2012). Therapist perspectives on community mental health services for children with autism spectrum disorders. Admin Pol Ment Health.

[9] Brookman-Frazee L, Taylor R, Garland AF (2010). Characterizing community-based mental health services for children with autism spectrum disorders and disruptive behavior problems. J Autism Dev Disord.

[10] Stahmer AC, Collings NM, Palinkas LA (2005). Early intervention practices for children with autism: descriptions from community providers. Focus Autism Other Dev Disabl..

[11] Forman SG, Shapiro ES, Codding RS, Gonzales JE, Reddy LA, Rosenfield SA (2013). Implementation science and school psychology. Sch Psychol Q.

[12] Dingfelder HE, Mandell DS (2011). Bridging the research-to-practice gap in autism intervention: an application of diffusion of innovation theory. J Autism Dev Disord.

[13] Aarons GA, Hurlburt M, Horwitz SM (2011). Advancing a conceptual model of evidence-based practice implementation in public service sectors. Admin Pol Ment Health.

[14] Aarons GA, Ehrhart MG, Torres E. Linking team level implementation leadership and implementation climate to individual level provider attitudes, behaviors, and implementation outcomes. Proceedings from the 8th Annual Conference on the Science of Dissemination & Implementation; Implement Sci. 2016;11(Suppl 2):100. 10.1186/s13012-016-0452-0. 10.1186/s13012-016-0452-0 PMC4977475 27490260

[15] Simonoff E, Pickles A, Charman T, Chandler S, Loucas T, Baird G (2008). Psychiatric disorders in children with autism spectrum disorders: prevalence, comorbidity, and associated factors in a population-derived sample. J Am Acad Child Adolesc Psychiatry.

[16] Mandell DS, Walrath CM, Manteuffel B, Sgro G, Pinto-Martin J (2005). Characteristics of children with autistic spectrum disorders served in comprehensive community-based mental health settings. J Autism Dev Disord.

[17] Brookman-Frazee L, Baker-Ericzén M, Stahmer A, Mandell D, Haine RA, Hough RL (2009). Involvement of youths with autism spectrum disorders or intellectual disabilities in multiple public service systems. J Ment Health Res Intellect Disabil.

[18] U.S. Department of Education, National Center for Education Statistics. Digest of Education Statistics: 2015, Chapter 2: Elementary and secondary education enrollments. National Center for Education Statistics. 2016. https://nces.ed.gov/programs/digest/d15/ch_2.asp. Accessed 8 Dec 2017.

[19] Leyfer OT, Folstein SE, Bacalman S, Davis NO, Dinh E, Morgan J (2006). Comorbid psychiatric disorders in children with autism: interview development and rates of disorders. J Autism Dev Disord.

[20] Horner RH, Carr EG, Strain PS, Todd AW, Reed HK (2002). Problem behavior interventions for young children with autism: a research synthesis. J Autism Dev Disord.

[21] National Autism Center. National Standards Project, Phase 2. National Autism Center; 2015. http://www.nationalautismcenter.org/national-standards-project/phase-2/. Accessed 8 Dec 2017.

[22] Wong C, Odom SL, Hume KA, Cox AW, Fettig A, Kucharczyk S (2015). Evidence-based practices for children, youth, and young adults with autism spectrum disorder: a comprehensive review. J Autism Dev Disord.

[23] Humphries TL (2003). Effectiveness of pivotal response training as a behavioral intervention for young children with autism spectrum disorders. Bridges: Practice-Based Research Syntheses.

[24] Odom SL, Collet-Klingenberg L, Rogers SJ, Hatton DD (2010). Evidence-based practices in interventions for children and youth with autism spectrum disorders. Prev Sch Fail.

[25] National Autism Center. National Standards Project, Phase 1 National Autism Center; 2009. http://www.nationalautismcenter.org/national-standards-project/history/. Accessed 8 Dec 2017.

[26] Simpson RL (2005). Evidence-based practices and students with autism spectrum disorders. Focus Autism Other Dev Disabl..

[27] Verschuur R, Didden R, Lang R, Sigafoos J, Huskens B (2013). Pivotal response treatment for children with autism spectrum disorders: a systematic review. Rev J Autism Dev Disord.

[28] Gengoux GW, Berquist KL, Salzman E, Schapp S, Phillips JM, Frazier TW (2015). Pivotal response treatment parent training for autism: findings from a 3-month follow-up evaluation. J Autism Dev Disord.

[29] Coolican J, Smith IM, Bryson SE (2010). Brief parent training in pivotal response treatment for preschoolers with autism. J Child Psychol Psychiatry.

[30] Pierce K, Schreibman L (1997). Multiple peer use of pivotal response training to increase social behaviors of classmates with autism: results from trained and untrained peers. J Appl Behav Anal.

[31] Stahmer AC (1995). Teaching symbolic play skills to children with autism using pivotal response training. J Autism Dev Disord.

[32] Pierce K, Schreibman L (1995). Increasing complex social behaviors in children with autism: effects of peer-implemented pivotal response training. J Appl Behav Anal.

[33] Brookman-Frazee L, Stahmer A, Baker-Ericzén MJ, Tsai K (2006). Parenting interventions for children with autism spectrum and disruptive behavior disorders: opportunities for cross-fertilization. Clin Child Fam Psychol Rev.

[34] Howard JS, Sparkman CR, Cohen HG, Green G, Stanislaw H (2005). A comparison of intensive behavior analytic and eclectic treatments for young children with autism. Res Dev Disabil.

[35] Suhrheinrich J, Stahmer AC, Schreibman L (2007). A preliminary assessment of teachers’ implementation of pivotal response training. J Speech Lang Pathol Appl Behav Anal.

[36] Suhrheinrich J, Stahmer AC, Reed S, Schreibman L, Reisinger E, Mandell D (2013). Implementation challenges in translating pivotal response training into community settings. J Autism Dev Disord.

[37] Montes G, Halterman JS, Magyar CI (2009). Access to and satisfaction with school and community health services for children with autism: national overview. Pediatrics.

[38] Magana S, Lopez K, Aguinaga A, Morton H (2013). Access to diagnosis and treatment services among Latino children with autism spectrum disorders. Intellect Dev Disabil..

[39] Magaña S, Parish SL, Rose RA, Timberlake M, Swaine JG (2012). Racial and ethnic disparities in quality of health care among children with autism and other developmental disabilities. Intellect Dev Disabil.

[40] Dyson MW, Chlebowski C, Wright BM, Brookman-Frazee L. Implementing a package of evidence-based strategies in community mental health programs for children with autism spectrum disorder: Therapist perspectives on training and delivery. under review.

[41] Stahmer AC, Suhrheinrich J, Reith S (2016). A pilot examination of the adapted protocol for classroom pivotal response teaching. Journal of the American Academy of Special Education Professionals.

[42] Brookman-Frazee L, Stahmer A, Stadnick N, Chlebowski C, Herschell A, Garland AF (2016). Characterizing the use of research-community partnerships in studies of evidence-based interventions in children’s community services. Admin Pol Ment Health.

[43] Wood JJ, McLeod BD, Klebanoff S, Brookman-Frazee L (2015). Toward the implementation of evidence-based interventions for youth with autism spectrum disorders in schools and community agencies. Behav Ther.

[44] Brookman-Frazee L, Drahota A (2010). An individualized mental health intervention for children with autism spectrum disorders (AIM HI): a model to address challenging behaviors in children with ASD—A therapist manual.

[45] Stahmer AC, Suhrheinrich J, Reed S, Schreibman L (2012). What works for you? Using teacher feedback to inform adaptations of pivotal response training for classroom use. Autism Res Treat.

[46] Brookman-Frazee LI, Drahota A, Stadnick N (2012). Training community mental health therapists to deliver a package of evidence-based practice strategies for school-age children with autism spectrum disorders: a pilot study. J Autism Dev Disord.

[47] Mandell DS, Stahmer AC, Shin S, Xie M, Reisinger E, Marcus SC (2013). The role of treatment fidelity on outcomes during a randomized field trial of an autism intervention. Autism.

[48] Strain PS, Bovey EH (2011). Randomized, controlled trial of the LEAP model of early intervention for young children with autism spectrum disorders. Topics Early Child Spec Educ..

[49] Beidas RS, Edmunds JM, Marcus SC, Kendall PC (2012). Training and consultation to promote implementation of an empirically supported treatment: a randomized trial. Psychiatr Serv.

[50] Chambers DA, Glasgow RE, Stange KC (2013). The dynamic sustainability framework: addressing the paradox of sustainment amid ongoing change. Implement Sci.

[51] Glisson C, Williams NJ (2015). Assessing and changing organizational social contexts for effective mental health services. Annu Rev Public Health.

[52] Grol RPTM, Bosch MC, Hulscher MEJL, Eccles MP, Wensing M (2007). Planning and studying improvement in patient care: the use of theoretical perspectives. Milbank Q.

[53] Lyon AR, Ludwig K, Romano E, Leonard S, Stoep AV, McCauley E (2013). “If it’s worth my time, I will make the time”: school-based providers’ decision-making about participating in an evidence-based psychotherapy consultation program. Admin Pol Ment Health.

[54] Aarons GA, Ehrhart MG, Farahnak LR, Hurlburt MS (2015). Leadership and organizational change for implementation (LOCI): a randomized mixed method pilot study of a leadership and organization development intervention for evidence-based practice implementation. Implement Sci.

[55] Bass BM, Avolio BJ (1990). The implications of transformational and transactional leadership for individual, team, and organizational development, in research in organizational change and development.

[56] Stogdill RM (1974). Handbook of leadership: a survey of theory and research.

[57] Edmondson AC (2003). Speaking up in the operating room: how team leaders promote learning in interdisciplinary action teams. J Manag Stud.

[58] Powell BJ, McMillen JC, Proctor EK, Carpenter CR, Griffey RT, Bunger AC (2012). A compilation of strategies for implementing clinical innovations in health and mental health. Med Care Res Rev.

[59] Novins DK, Green AE, Legha RK, Aarons GA (2013). Dissemination and implementation of evidence-based practices for child and adolescent mental health: a systematic review. J Am Acad Child Adolesc Psychiatry.

[60] Green AE, Albanese BJ, Shapiro NM, Aarons GA (2014). The roles of individual and organizational factors in burnout among community-based mental health service providers. Psychol Serv.

[61] Glisson C, Hemmelgarn A, Green P, Williams NJ (2013). Randomized trial of the availability, responsiveness and continuity (ARC) organizational intervention for improving youth outcomes in community mental health programs. J Am Acad Child Adolesc Psychiatry.

[62] Williams NJ, Glisson C (2014). Testing a theory of organizational culture, climate and youth outcomes in child welfare systems: a United States national study. Child Abuse Negl.

[63] Schein EH (2010). Organizational culture and leadership.

[64] Priestland A, Hanig R (2005). Developing first-level leaders. Harv Bus Rev.

[65] Klein KJ, Conn AB, Smith DB, Sorra JS (2001). Is everyone in agreement? An exploration of within-group agreement in employee perceptions of the work environment. J Appl Psychol.

[66] Casper ES (2007). The theory of planned behavior applied to continuing education for mental health professionals. Psychiatr Serv.

[67] Henggeler SW, Chapman JE, Rowland MD, Halliday-Boykins CA, Randall J, Shackelford J (2008). Statewide adoption and initial implementation of contingency management for substance-abusing adolescents. J Consult Clin Psychol.

[68] Aarons GA (2004). Mental health provider attitudes toward adoption of evidence-based practice: the evidence-based practice attitude scale (EBPAS). Ment Health Serv Res.

[69] Reding ME, Chorpita BF, Lau AS, Innes-Gomberg D (2014). Providers’ attitudes toward evidence-based practices: is it just about providers, or do practices matter, too?. Admin Pol Ment Health.

[70] Augustsson H, von Thiele SU, Stenfors-Hayes T, Hasson H (2014). Investigating variations in implementation fidelity of an organizational-level occupational health intervention. International Journal of Behavioral Medicine.

[71] Beidas RS, Edmunds J, Ditty M, Watkins J, Walsh L, Marcus S (2014). Are inner context factors related to implementation outcomes in cognitive-behavioral therapy for youth anxiety?. Admin Pol Ment Health.

[72] Lim A, Nakamura BJ, Higa-McMillan CK, Shimabukuro S, Slavin L (2012). Effects of workshop trainings on evidence-based practice knowledge and attitudes among youth community mental health providers. Behav Res Ther.

[73] Harned MS, Dimeff LA, Woodcock EA, Kelly T, Zavertnik J, Contreras I (2014). Exposing clinicians to exposure: a randomized controlled dissemination trial of exposure therapy for anxiety disorders. Behav Ther.

[74] Harned MS, Dimeff LA, Woodcock EA, Contreras I (2013). Predicting adoption of exposure therapy in a randomized controlled dissemination trial. J Anxiety Disord.

[75] Allen KA, Bowles TV (2014). Examining the effects of brief training on the attitudes and future use of behavioral methods by teachers. Behav Interv.

[76] McCormick JA (2011). Inclusive elementary classroom teacher knowledge of and attitudes toward applied behavior analysis and autism Spectrum disorder and their use of applied behavior analysis [doctoral dissertation].

[77] Henman MJ, Butow PN, Brown RF, Boyle F, Tattersall MHN (2002). Lay constructions of decision-making in cancer. Psychooncology.

[78] Jahng KH, Martin LR, Golin CE, DiMatteo MR (2005). Preferences for medical collaboration: patient–physician congruence and patient outcomes. Patient Educ Couns.

[79] Kaplan SH, Greenfield S, Ware JE (1989). Assessing the effects of physician-patient interactions on the outcomes of chronic disease. Med Care.

[80] Ong LML, de Haes JCJM, Hoos AM, Lammes FB (1995). Doctor-patient communication: a review of the literature. Soc Sci Med.

[81] Stewart M, Brown JB, Donner A, McWhinney IR, Oates J, Weston WW (2000). The impact of patient-centered care on outcomes. J Fam Pract.

[82] Trummer UF, Mueller UO, Nowak P, Stidl T, Pelikan JM (2006). Does physician–patient communication that aims at empowering patients improve clinical outcome? A case study. Patient Educ Couns.

[83] Street RL, Piziak VK, Carpentier WS, Herzog J, Hejl J, Skinner G (1993). Provider-patient communication and metabolic control. Diabetes Care.

[84] Magnus M, Jones K, Phillips G, Binson D, Hightow-Weidman LB, Richards-Clarke C (2010). Characteristics associated with retention among African American and Latino adolescent HIV-positive men: results from the outreach, care, and prevention to engage HIV-seropositive young MSM of color special project of National Significance Initiative. J Acquir Immune Defic Syndr.

[85] Street RL, Gordon H, Haidet P (2007). Physicians' communication and perceptions of patients: is it how they look, how they talk, or is it just the doctor?. Soc Sci Med.

[86] Rubak S, Sandbæk A, Lauritzen T, Christensen B (2005). Motivational interviewing: a systematic review and meta-analysis. Br J Gen Pract.

[87] Nock MK, Kazdin AE (2005). Randomized controlled trial of a brief intervention for increasing participation in parent management training. J Consult Clin Psychol.

[88] Miller CE, Johnson JL (2001). Motivational interviewing. Can Nurse.

[89] Rakovshik SG, McManus F (2010). Establishing evidence-based training in cognitive behavioral therapy: a review of current empirical findings and theoretical guidance. Clin Psychol Rev.

[90] Odom SL (2009). The tie that binds: evidence-based practice, implementation science, and outcomes for children. Topics Early Child Spec Educ.

[91] Scheuermann B, Webber J, Boutot EA, Goodwin M (2003). Problems with personnel preparation in autism spectrum deisorders. Focus Autism Other Dev Disabl.

[92] Miller WR, Yahne CE, Moyers TB, Martinez J, Pirritano M (2004). A randomized trial of methods to help clinicians learn motivational interviewing. J Consult Clin Psychol.

[93] Sholomskas DE, Syracuse-Siewert G, Rounsaville BJ, Ball SA, Nuro KF, Carroll KM (2005). We don't train in vain: a dissemination trial of three strategies of training clinicians in cognitive-behavioral therapy. J Consult Clin Psychol.

[94] Beidas RS, Kendall PC (2010). Training therapists in evidence-based practice: a critical review of studies from a systems-contextual perspective. Clin Psychol Sci Pr.

[95] National Research Council (2001). Educating children with autism.

[96] Bush RN (1984). Effective staff development.

[97] Cornett J, Knight J, Knight J (2009). Research on coaching. Coaching: approaches and perspectives.

[98] Aarons GA, Ehrhart MG, Farahnak LR (2014). The implementation leadership scale (ILS): development of a brief measure of unit level implementation leadership. Implement Sci.

[99] Ehrhart MG, Aarons GA, Farahnak LR. Assessing the organizational context for EBP implementation: the development and validity testing of the Implementation Climate Scale (ICS). Implementation Science : IS. 2014;9:157. 10.1186/s13012-014-0157-1. 10.1186/s13012-014-0157-1 PMC4210525 25338781

[100] Seal KH, Abadjian L, McCamish N, Shi Y, Tarasovsky G, Weingardt K (2012). A randomized controlled trial of telephone motivational interviewing to enhance mental health treatment engagement in Iraq and Afghanistan veterans. Gen Hosp Psychiatry.

[101] Westra HA, Arkowitz H (2011). Special series: integrating motivational interviewing with cognitive behavioral therapy for a range of mental health problems. Cogn Behav Pract.

[102] Naar S, Flynn H, Arkowitz H, Miller W, Rollnick S (2015). Motivational interviewing and the treatment of depression. Motivational interviewing in the treatment of psychological problems.

[103] Brown CH, Wang W, Kellam SG, Muthén BO, Petras H, Toyinbo P (2008). Methods for testing theory and evaluating impact in randomized field trials: intent-to-treat analyses for integrating the perspectives of person, place, and time. Drug Alcohol Depend.

[104] Enders CK (2011). Missing not at random models for latent growth curve analyses. Psychol Methods.

[105] Diggle P, Kenward MG (1994). Informative drop-out in longitudinal data analysis. J R Stat Soc Ser C Appl Stat.

[106] Little RJA, Rubin DB (2002). Statistical analysis with missing data 2nd ed.

[107] Preacher KJ, Curran PJ, Bauer DJ (2006). Computational tools for probing interactions in multiple linear regression, multilevel modeling, and latent curve analysis. J Educ Behav Stat.

[108] MacKinnon DP (2008). Introduction to statistical mediation analysis.

[109] Hedeker D, Gibbons RD, Waternaux C (1999). Sample size estimation for longitudinal designs with attrition: comparing time-related contrasts between two groups. J Educ Behav Stat.

[110] Thoemmes F, MacKinnon DP, Reiser MR (2010). Power analysis for complex mediational designs using Monte Carlo methods. Struct Equ Model Multidiscip J.

[111] Palinkas LA, Schoenwald SK, Hoagwood K, Landsverk J, Chorpita BF, Weisz JR (2008). An ethnographic study of implementation of evidence-based treatments in child mental health: first steps. Psychiatr Serv.

[112] Garland AF, Hurlburt MS, Brookman-Frazee L, Taylor RM, Accurso EC (2010). Methodological challenges of characterizing usual care psychotherapeutic practice. Admin Pol Ment Health.

[113] Curran GM, Bauer M, Mittman B, Pyne JM, Stetler C (2012). Effectiveness-implementation hybrid designs: combining elements of clinical effectiveness and implementation research to enhance public health impact. Med Care.

[114] Drahota A, Stadnick N, Brookman-Frazee L (2014). Therapist perspectives on training in a package of evidence-based practice strategies for children with autism spectrum disorders served in community mental health clinics. Admin Pol Ment Health.

[115] Leeman J, Calancie L, Kegler MC, Escoffery CT, Herrmann AK, Thatcher E, et al. Developing theory to guide building practitioners' capacity to implement evidence-based interventions. Health Educ Behav. 2015; 10.1177/1090198115610572. 10.1177/1090198115610572 PMC5330318 26500080

[116] Nock MK, Photos V (2006). Parent motivation to participate in treatment: assessment and prediction of subsequent participation. J Child Fam Stud.

[117] Ehrhart MG, Aarons GA, Farahnak LR (2014). Assessing the organizational context for EBP implementation: the development and validity testing of the implementation climate scale (ICS). Implement Sci.

